# Who needs the social model of disability?

**DOI:** 10.3389/fsoc.2023.1305301

**Published:** 2023-12-07

**Authors:** Sofia Adam, Athanasios Koutsoklenis

**Affiliations:** ^1^Department of Social Policy, Democritus University of Thrace, Komotini, Greece; ^2^Department of Primary Education, Democritus University of Thrace, Komotini, Greece

**Keywords:** disability, disabled, social model of disability, disability studies, critical disability studies

## Abstract

Over the past two decades, there has been a growing shift away from the Social Model of Disability (SMD) in both theory and practice. This article aims to substantiate the relevance of SMD by addressing the main arguments against it and by identifying why and for whom it is still relevant. In the introductory section, we focus on the recent production of multiple disability models in order to contextualize their emergence and elucidate the reasons behind their proliferation. In the main section of the article, we critically engage with three lines of criticism against SMD in order to explain why it remains relevant and for whom. Our main point is that, in the context of the neoliberal capitalist era, the SMD is indispensable for all disabled persons who have been denied their dignity, both in material and cultural terms.

## Introduction: why so many disability models?

The Social Model of Disability (SMD) builds on the fundamental principles of disability developed by the Union of the Physically Impaired Against Segregation (UPIAS), a union of disabled activists in the UK in the mid 70s (Union of the Physically Impaired Against Segregation, 1976). SMD, a term coined by Oliver (1982), changed fundamentally the theorization of disability by diverting the analytical focus from the individual to the social level and by stressing the distinction between impairment and disability. In a nutshell, “disability is the disadvantage or restriction of activity caused by the political, economic and cultural norms of a society which takes little or no account of people who have impairments and thus excludes them from mainstream activity (Therefore disability, like racism or sexism, is discrimination and social oppression). Impairment is a characteristic of the mind, body or senses within an individual which is longterm and may, or may not, be the result of disease, genetics or injury” (Oliver et al., 2012, p.16).

Already in the 1990s, calls for the “revision” of the SMD emerged (Finkelstein, 2001, 2007). From the first decade of the 2000s onwards, critics not only called for a revision of SMD but also for its rejection. These calls are numerous and have appeared incessantly in the literature since then (e.g., Bury, 1997; Shakespeare, 2004, 2006, 2014; Riddle, 2020). The quests for revision and/or rejection go hand in hand with the proliferation of disability models to the extent that the taxonomy of disability models has become a scientific project in its own right.^1^

Disability models are conceptual devices based on implicit and/or explicit theoretical premises aiming at (a) the conceptualization of impairment, (b) the conceptualization of disability, (c) the identification of the problem, and d) the organization of responses to the problem (Karagianni and Koutsoklenis, 2023). Following this definition, the proliferation of disability models is based on the selection of arbitrary criteria for classification and/or the sublimation of disparate approaches to the level of a model. As a result, the proposed taxonomies are not often scientifically robust because they do not expose the methodological criteria according to which they differentiate among the various models. For instance, there is a confusion between models and historical stages of the perception of disability (see, for example, the taxonomy proposed by Goodley, 2011) or arbitrary distinctions such as that between the social and the economic model of disability (see, for example, the taxonomy proposed by Retief and Letšosa, 2018).

However, the very tendency to produce disability models occurs on the grounds of broader social transformations. To our understanding, the proliferation of disability models is a by-product of recent developments in social organization and their crystallization in academic discourse. Interestingly, it was the SMD that elucidated the interplay between social organization and impairment and how this interplay results in disability. In particular, SMD explicitly focused on how dominant social structures and practices produce and perpetuate disability in mobility, education, health care, etc. (Oliver, 1996). These developments took place in a context of new social movements—in parallel with the feminist and environmentalist movements—articulating collectively their demands in order to change the social structures producing and perpetuating disability (Oliver, 1997). The purpose of the disability movement was to be “consciously engaged in critical evaluation of capitalist society and in the creation of alternative models of social organization at local, national and international levels, as well as trying to reconstruct the world ideologically and to create alternative forms of service provision” (Oliver, 1990, p.113).

SMD heavily influenced the formation of the disability studies as an academic discipline on its own within which disabled researchers explored the many facets of disability (i.e., aging, ethnicity and “race”, and sexuality; Barnes, 2020). Ironically, it was exactly this opening up of the interplay between social organization and disability which was conducive to the recent proliferation of new disability models. In other words, the proliferation of disability models emerges in the fragmented context of late capitalism (see for the meaning of late capitalism, Jameson, 1992) where both collective horizon and the analytical focus on social structures have receded.

## The social model of disability and its discontents revisited

The proliferation of disability models has, to a significant extent, discredited SMD by reducing it to a caricature, stripping away its core meaning. There are several lines of criticism. In the following, we will focus on three types of criticism: (a) the SMD does not take into account the experience of impairment, (b) it is not inclusive of the various types of impairment, and (c) it does not challenge the distinction between the disabled and non-disabled because of its adherence to modernist binary thinking.

The first line of criticism highlights that SMD ignores the experience of impairment (e.g., Hughes, 2009; Anastasiou and Kauffman, 2013). For instance, referring to the proponents of SMD.^2^ Anastasiou and Kauffman (2013, p. 446)^3^ argue that “…when biological or intrinsic characteristics are neglected as a reality, disability becomes a neutral thing-something we do not really care about one way or the other”. They go on to stress that “by choosing to theorize only on sociological grounds, they detach biological and mental elements from the disabled subject” (Anastasiou and Kauffman, 2013, p.445). If the proponents of SMD devalued the experience of impairment, they would not engage in research and theory about it. Oliver himself went on to co-author two books about the experience of impairment, with the characteristic titles *Walking into darkness: The experience of spinal cord injury* (Oliver et al., 1988) and *What do they expect after all these years? Aging with a disability* (Zarb and Oliver, 1993). The difference with contemporary critics of SMD is the analytical focus of the engagement with the experience of impairment. According to the SMD, the experience of impairment is not limited solely to the level of individual psychology or interpersonal relationships (Oliver, 1990). Instead, it encompasses a wide range of social and material manifestations, such as family status, income, education, work and so on (Barnes and Mercer, 2006). In brief, the basic contribution of SMD is that personal experiences of impairment can be represented and understood in ways which enable collective rather than individual trajectories, and the politicization of disablement (Oliver, 1990, 1997, 2004; Finkelstein, 1996, 2007). This was not an uncritical position but a deliberate choice against a historical interpretation of impairment that led to the tradition of “compassionate biography” (Hunt, 1966).

Regarding the different types of impairment, the criticism highlights that the SMD was mainly developed by people with physical impairments, such as Vic Finkelstein, Paul Hunt, and Mike Oliver. Therefore, it is not suitable for people with other types of impairments, who should preferably organize themselves according to their specific type of impairment (e.g., Shakespeare, 2014; Woods, 2017; Anderson-Chavarria, 2021). Proponents of the SMD see little value in impairment-specific organization which is limited to the needs and types of support related to impairment effects (Oliver and Barnes, 2012). Instead, they insist that the priority should be the analysis of the material and socio-political forces that cause disability; these forces are common and independent of the type of impairment (Oliver and Barnes, 2012). After all, the focus on the particular types of impairment was one of the major barriers in attempts to organize collectively a movement of the disabled up to the 70s (Campbell and Oliver, 1996) whereas these differences were used strategically in the neoliberal backlash: “Our differences are used to slash our services as our needs are now being assessed as being moderate, substantial or critical and many local authorities are now only providing services to those whose needs are critical. The disabled peoples' movement that was once united around the barriers we had in common now faces deep divisions and has all but disappeared, leaving disabled people at the mercy of an ideologically driven government with no-one to defend us except the big charities who are driven by self-interest” (Oliver, 2013, p.3). In addition, there are more reasons to question the re-emerging focus on types of impairment in the neoliberal context with a proliferation of diagnostic classifications in need of market solutions (Kirk et al., 2013).^4^

Critical Disability Studies (CDS) pose a far more intriguing challenge toward SMD. They develop postconventional approaches to disability which stress the “significance of embodiment; an awareness of the workings of the cultural imaginary; a deconstruction of binary thought in favor of the fluidity of all categories; and a recognition that emotion and affect are as important as the material aspects of life” (Shildrick, 2020, p.34). According to this framework^5^, the category of the disabled is contested as it is itself subject to binary thinking, an ill inherited from modernity. Following the work of scholars who engaged critically with both disability and feminist theory (Garland-Thomson, 1997, 2002), this approach elucidates the constantly changing types of impairment included in the category of the disabled as well as the “intersectional concerns—such as those of ethnicity, age, class, sexuality, gender, and more—that impact on the experience and significance of any disabled state” (Shildrick, 2020, p. 35). These insights are targeted against the Social Constructionist Model of Disability which insists “that the major “problem” of disability is located not in the marginalized individual but within the normative structures of mainstream society” (Shildrick, 2020, p.37). CDS insist that the Social Constructionist Model restricts the political horizon in formal structures of equality without challenging the normativity of these structures, addresses inadequately the question of agency and treats disability as only a problem of material exclusion. Instead, the task of CDS is to open up for all “regardless of our individual morphology” the responsibility to interrogate the “sociocultural imaginary that pervasively shapes the disposition of everyday attitudes and values” (Shildrick, 2020, p.38–39). More importantly, there is a weightier responsibility on those who are externally defined as non-disabled to interrogate their “cultural and psychosocial location as non-disabled” (Shildrick, 2020, p.39).

It is not clear whether CDS in the preceding analysis target SMD or not. We are not certain if the choice to differentiate between SMD and the Social Constructionist Models of Disability is a consistent one especially in light of the initial statement that CDS intend to enrich the SMD by expanding its analytical rigor. The Social Constructionist Model of thus presented understands disability mainly as a problem of discrimination to be rectified by the extension of human rights to those pre-defined as disabled and as a quest for material gains. If that is the case, the Social Constructionist Model is not equated with SMD for a number of reasons.

First, the SMD by placing the analytical interest on the social structures and practices leading to disablement, enables the identification of common causes at the societal level. In this way, the SMD defies the binary between materialism and discourse that has been kept intact, to our puzzlement, in much of the postconventional approaches to disability in clear distance from similar attempts to conceptualize gender in a post-structural fashion (Butler, 1998). On the one hand, the discursive is never merely cultural but enmeshed in the political economy of production and reproduction of human life. On the other hand, the material does not refer only to the body and/or the built environment, as something tangible. Our current society operates under the capitalist law of value. Its basic tenets, abstract labor, use-value and exchange-value, are not tangible but they are extremely material in the type of social life they produce. Instead of a juxtaposition between the cultural and the material which seems to trouble attempts to reconceptualize disability (i.e., Garland-Thomson, 2011), we argue that there is a dialectic unity of materiality and discourse. That is the question of disability is neither simply material nor simply discursive, but social in its dialectic unity.

Second, the SMD by treating disability as the outcome of disabling social factors is in clash with a subjectivation along additive fixed identities. Who is to be included or not is the outcome of a movement formation whose participants object to these societal factors as the root causes of their state as disabled. Therefore, the subject is not a pre-defined category waiting to be empowered, but the outcome of the agency involved in exactly identifying the root causes of the state of disablement. From this perspective, both the boundaries of the disabled as well as the identification of the root causes are constantly contested not only externally but also internally as the result of the agency involved in collective action.

Third, the postconventional approaches do acknowledge the strategic significance of SMD but at the same time undermine its premises. Allegedly, SMD addresses only the distribution of rights and resources without challenging the dominant normative conceptualization of disability. We consider that this reading does not do justice to the historical developments taking place within the disability movement and as result of the framing enabled by SMD. The disability movement not only demanded access to rights and resources, but also enabled the organization of alternative service provision in a prefigurative manner challenging dominant narratives about service provision for the disabled (Finkelstein, 2007; Oliver and Barnes, 2012; Barnes, 2020).

Fourth, the postconventional approaches by inviting each one of us to interrogate the sociocultural imaginary and the binary disabled/non-disabled do not take into account the extremely varied positions in terms of access to power and resources still existing in our neoliberal capitalist society. In other words, not all (disabled and non-disabled alike), and especially each one of us left alone, possess the social, economic and political resources (time included) to reflect on their status and interrogate dominant assumptions about disability. More importantly, especially the ones who are externally identified as non-disabled and, in the position to design and implement policies affecting others, will not interrogate their standards and privileges if they are left unchallenged. However, this endeavor necessitates the collective action of those affected and in need of changing both dominant discourses and policies. All those who still need SMD.

## Discussion

The main argument of this article is exemplified by the juxtaposition between two movies: “The Intouchables” (directors Olivier Nakache and Éric Toledano, 2011)^6^ and “I, Daniel Blake” (director Ken Loach, 2016).^7^ The first movie depicts the evolving and transformative bond between Philippe, a wealthy individual paralyzed from the neck down after a paragliding accident, and his caregiver, Driss, a black man who hails from the Parisian ghettos. Driss initially seeks the job just to obtain a signature required to continue receiving his unemployment benefits.^8^ Surprisingly, Philippe hires him, and in the process, Philippe discovers new interests in diverse music genres and marijuana through Driss. In summary, reproducing all types of racial stereotypes and cliches, the movie is pleasing in highlighting the benefits of companionship, affection and personal services. The second film revolves around the life of Daniel Blake, a 59-year-old man who, following a heart attack, resolves to challenge the government's decision regarding his employment and support allowance. As described by Peter (2021), Dan's determination to secure his benefits is consistently thwarted. Being unfamiliar with computers, he must seek assistance from multiple individuals just to complete a basic online appeal form. Additionally, he spends hours waiting on hold to speak with government representatives, only to be told that they cannot assist him and refer him to other representatives. In this demoralizing cycle, Dan becomes entangled in dense bureaucratic language that appears deliberately confusing. None of the people he interacts with can provide clear answers; they simply defer to “decision makers” who will determine Dan's fate without ever meeting him. What is even worse, Dan is persuaded to apply for unemployment benefits despite his doctor's assessment of his unfitness for work, solely because a federal test suggested otherwise.^9^

Watching these two movies from the perspective of disability studies exemplifies why and for whom the SMD is still relevant. Philippe can benefit from the personal services through his private means. A lot can be discerned about the significance of care as a relational practice defying the roles of giver and receiver. However, this option is not available for Daniel. Daniel has to struggle through the undercut and outcontracted public services in order to access resources to make a living. In this process, he is also a giver toward a young unemployed single mother. Care is relational again but more in terms of solidarity and in becoming a quest for a policy change.

The lines of criticism to the SMD presented in this paper do not suffice to revise, let alone, replace it. They fall prey to an essentialism (i.e., the essence of impairment defines disability) which opens up the room for further medicalization especially in the context of neoliberal capitalism (Honkasilta and Koutsoklenis, 2022). They undermine the gravity of barriers along gender, age, sexual orientation by inserting them into the cultural as opposed to the material. They fall short of opening a perspective enabling collective action for and by all those denied their dignity, in material and cultural terms.

The political value of SMD has been recently confirmed. In the wake of the global capitalist crisis, a series of official and unofficial policies were drawn up and implemented, including—among others—reductions in benefits, curtailment of services and the “demonisation” of disabled people (Karagianni, 2017; Ryan, 2019). Others have also suggested the relevance of SMD but with a strong emphasis on its potential to “enable” and “ensure” human rights (Berghs et al., 2019). We deem SMD significant and relevant exactly because it opens up the route for collective demands and welfare claims without losing sight of the disabling practices of welfare state institutions. But for such claims to be reasserted today, we have to be clear about whom they matter for, why they matter, what would be the appropriate mix of benefits and services and how these should be organized.

## Data availability statement

The original contributions presented in the study are included in the article/supplementary material, further inquiries can be directed to the corresponding author.

## Author contributions

SA: Writing—original draft, Writing—review & editing. AK: Writing—original draft, Writing—review & editing.

## Footnotes

^1^An indicative list of self-proclaimed disability models includes: individual-medical, social, minority, cultural, relational, moral, consumer, economic, human rights, diversity, critical disability studies, systemic analysis, identity, charity, religious, limits, rehabilitation (Karagianni and Koutsoklenis, 2023).

^2^Anastasiou and Kauffman (2013) choose to refer to proponents of the SMD as “social constructionists”. Interestingly, the same term is adopted by proponents of postconventional approaches as will be explained.

^3^We chose here to engage with the work of Anastasiou and Kauffman (2013) because we deem it representative of the position under scrutiny.

^4^The critical engagement with the proliferation of diagnostic classifications related to impairment is beyond the scope of this paper.

^5^We chose here to engage with the work of Shildrick (2020) published in the influential Routledge Handbook of Disability Studies because it manages to comprehensively encapsulate the postconventional approaches.

^6^ https://www.imdb.com/title/tt1675434/

^7^ https://www.imdb.com/title/tt5168192/?ref_=fn_al_tt_1

^8^The movie reproduces, apart from racial stereotypes and cliches, the classical polemic against welfare claimants as persons who survive on welfare benefits without any interest to work resembling the main narrative of workfare strategies in social policy.

^9^ https://independent-magazine.org/2021/06/14/i-daniel-blake-review/
